# Advanced endoscopic interventions on the pancreas and pancreatic ductal system: a primer for radiologists

**DOI:** 10.1186/s13244-019-0689-7

**Published:** 2019-01-28

**Authors:** Massimo Tonolini, Emilia Bareggi, Pietro Gambitta

**Affiliations:** 1 Department of Radiology, “Luigi Sacco” University Hospital, Via G.B. Grassi 74, 20157 Milan, Italy; 2 Digestive Endoscopy, “Luigi Sacco” University Hospital, Via G.B. Grassi 74, 20157 Milan, Italy; 3 Present address: Head, Department of Gastroenterology / Digestive Endoscopy, New Hospital of Legnano, Via Papa Giovanni Paolo II, 20025 Legnano, Italy

**Keywords:** Pancreas, Pancreatitis, Pancreatic pseudocyst, Pancreatic duct, Endoscopy, Computed tomography (CT), Magnetic resonance imaging (MRI)

## Abstract

In recent years, technological advancements including endoscopic ultrasound (EUS) guidance and availability of specifically designed stents further expanded the indications and possibilities of interventional endoscopy. Although technically demanding and associated with non-negligible morbidity, advanced pancreatic endoscopic techniques now provide an effective minimally invasive treatment for complications of acute and chronic pancreatitis.

Aiming to provide radiologists with an adequate familiarity, this pictorial essay reviews the indications, techniques, results and pre- and post-procedural cross-sectional imaging appearances of advanced endoscopic interventions on the pancreas and pancreatic ductal system. Most of the emphasis is placed on multidetector CT and MRI findings before and after internal drainage of pseudocysts and walled-off necrosis via EUS-guided endoscopic cystostomy, and on stent placement to relieve strictures or disruption of the main pancreatic duct, respectively in patients with chronic pancreatitis and disconnected pancreatic duct syndrome.

## Key points

- In recent years, technological advancements including endoscopic ultrasound (EUS) guidance and specifically designed stents expanded the indications and possibilities of interventional pancreatic endoscopy.
- Although technically demanding and associated with non-negligible morbidity, advanced pancreatic endoscopy now provides an effective minimally invasive treatment for complications of acute and chronic pancreatitis.
- State-of-the art cross-sectional imaging with multidetector CT and MRI plays a pivotal role in the planning of endoscopic pancreatic interventions, assessment of efficacy and timely detection of complications.
- EUS-guided endoscopic cystostomy and necrosectomy now represent the preferred treatment option to achieve internal drainage of pseudocysts, walled-off pancreatic necrosis and infected collections into the digestive tract.
- Endoscopic stenting of the main pancreatic duct is indicated to manage benign, postsurgical and selected malignant strictures, most usually in patients with chronic pancreatitis and disconnected pancreatic duct syndrome.

## Introduction

Over the last two decades, interventional endoscopy progressively established itself as the primary therapeutic modality for the majority of spontaneous and postoperative disorders affecting the biliary tract and pancreas. In recent years, technological advancements such as endoscopic ultrasound (EUS) guidance and availability of novel, specifically designed stents further expanded the indications and possibilities of interventional endoscopy. Focusing on the pancreas, endoscopic techniques increasingly provide effective, minimally invasive treatment for complications of acute and chronic pancreatitis including internal drainage of collections and management of main pancreatic duct (MPD) stricture or disruption. Albeit it generally obviates the need for surgery, advanced pancreatic endoscopy remains technically demanding and requires special training: moreover, the increasing complexity of procedures carries a substantial risk of post-procedural morbidity [1, 2].

Nowadays, multidetector CT (MDCT) and MRI including MR-cholangiopancreatography (MRCP) are widely used to investigate patients with pancreatic disorders before and after surgical or interventional procedures. This pictorial essay presents indications, techniques, expected and abnormal cross-sectional imaging appearances of advanced endoscopic interventions on the pancreas and pancreatic ductal system [3].

### Pre-procedural MDCT and MRI imaging

#### Complications of acute pancreatitis

In the vast majority of situations, contrast-enhanced MDCT is the mainstay modality for the diagnosis and staging of acute pancreatitis (AP). In recent years, technical advancements in MRI such as multichannel phased-array coils, parallel imaging, respiratory-triggered and navigator-echo-based techniques allowed for faster acquisition, improved spatial resolution and decreased need for patient cooperation, thereby resulting in an increased use of MRI to investigate patients with acute abdominal disorders such as AP [4].

**Fig. 1:**
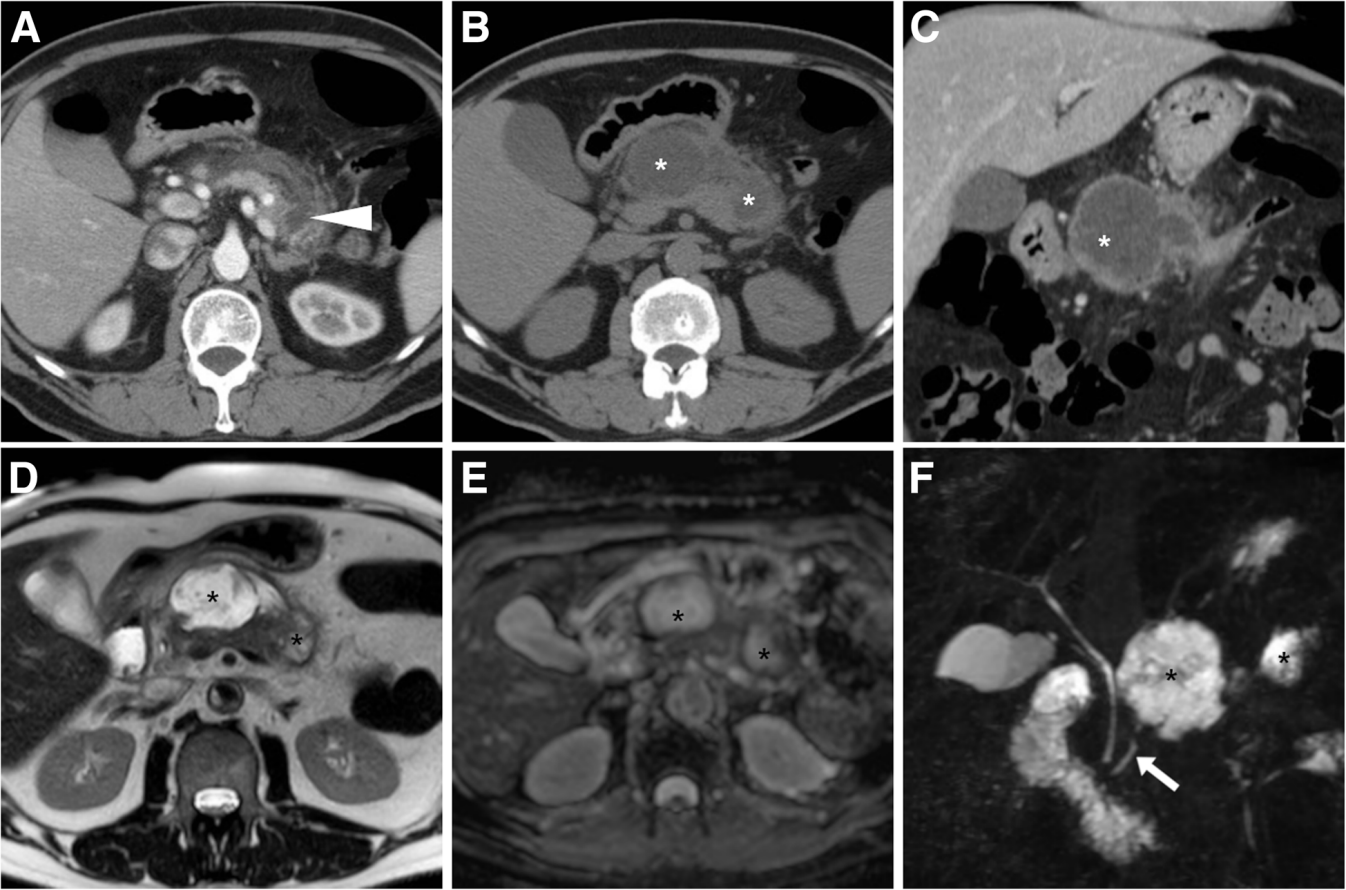
In a 55-year-old male with clinical and laboratory diagnosis of acute pancreatitis (AP), initial contrast-enhanced multidetector CT (MDCT, **a**) showed mild peripancreatic oedema and a limited region of nonenhancing parenchyma (arrowhead) at the pancreatic tail. Follow-up unenhanced (**b**) and post-contrast (**c**) CT showed development of a bilobated hypoattenuating collection (*) in the lesser sac. A month later, MRI confirmed diagnosis of non-infected walled-off pancreatic necrosis (WOPN) by showing persistent collection (*) with internal low-signal debris on T2-weighted images (**d**), without restricted diffusion on apparent diffusion coefficient (ADC) map (**e**). Note superimposition of collection (*) on the main pancreatic duct (MPD, arrow) on MR-cholangiopancreatography (MRCP) image (**f**)

**Fig. 2:**
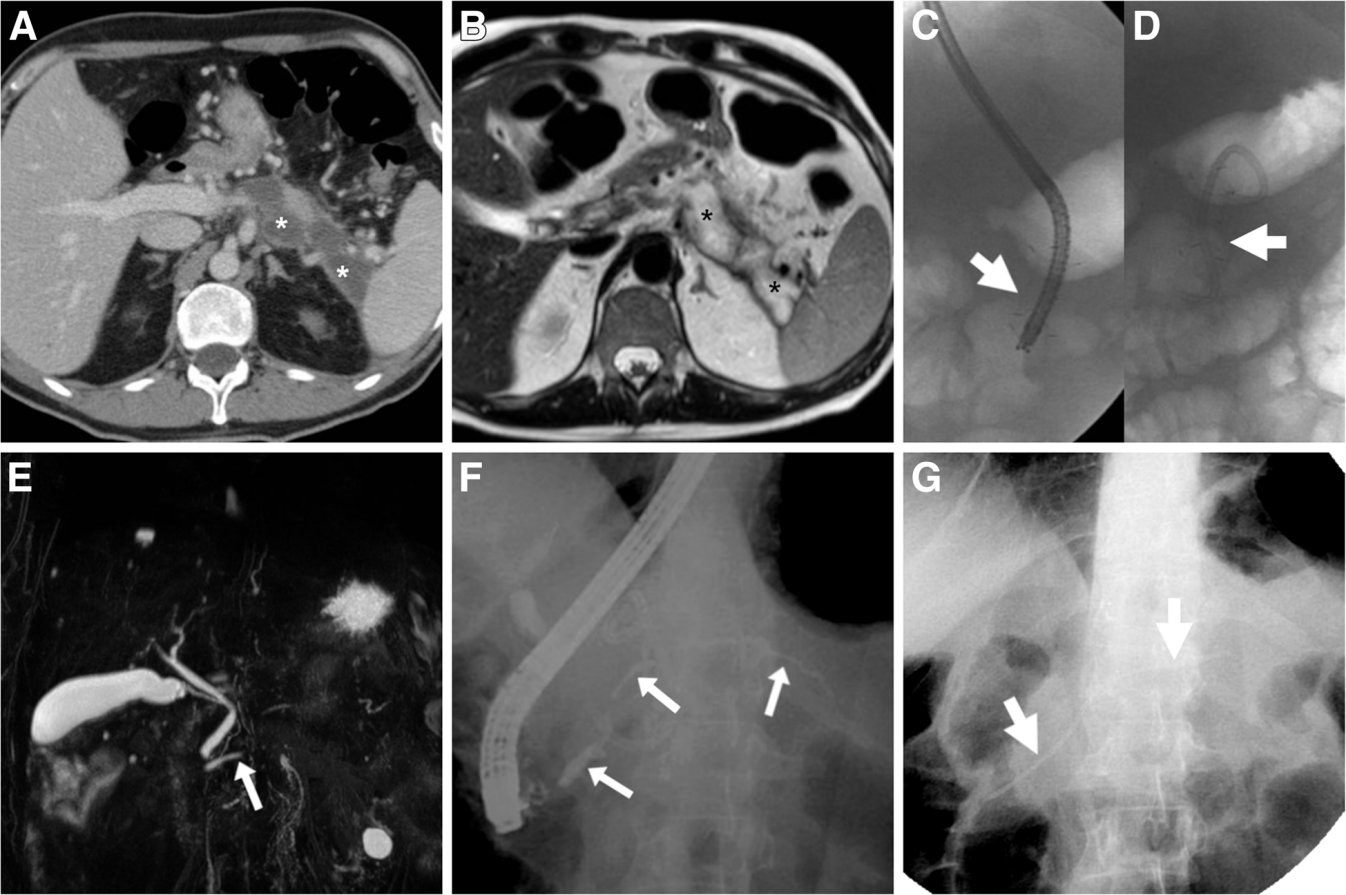
In a 56-year-old male, a year after severe necrotic-haemorrhagic AP, T2-weighted MRI (**a**) and contrast-enhanced MDCT (**b**) showed persistent ample regions of hyperintense, nonenhancing parenchymal necrosis (*). At another hospital, endoscopic ultrasound (EUS)-guided cystogastrostomy was initially performed, including positioning of a 3-cm-long, 12-mm-wide self-expanding metal stent (thick arrow in **c**). After obtaining incomplete regression of necrotic collection, repeated endoscopy at our institution included trans-stent necrosectomy and deployment of a plastic pigtail stent (thick arrow in **d**). After poor, prolonged clinical improvement, MRCP (**e**) showed mild MPD dilatation at the pancreatic head (arrow) and segmental discontinuity at the body. Diagnosis of disconnected pancreatic duct syndrome (DPDS) was confirmed during endoscopic retrograde cholangiopancreatography (ERCP, **f**) and definitively treated by positioning of a long stent (thick arrows in **g**) through the disrupted MPD, ultimately resulting in relieved complaints and laboratory changes (adapted from Open Access ref. no. [27])

**Fig. 3:**
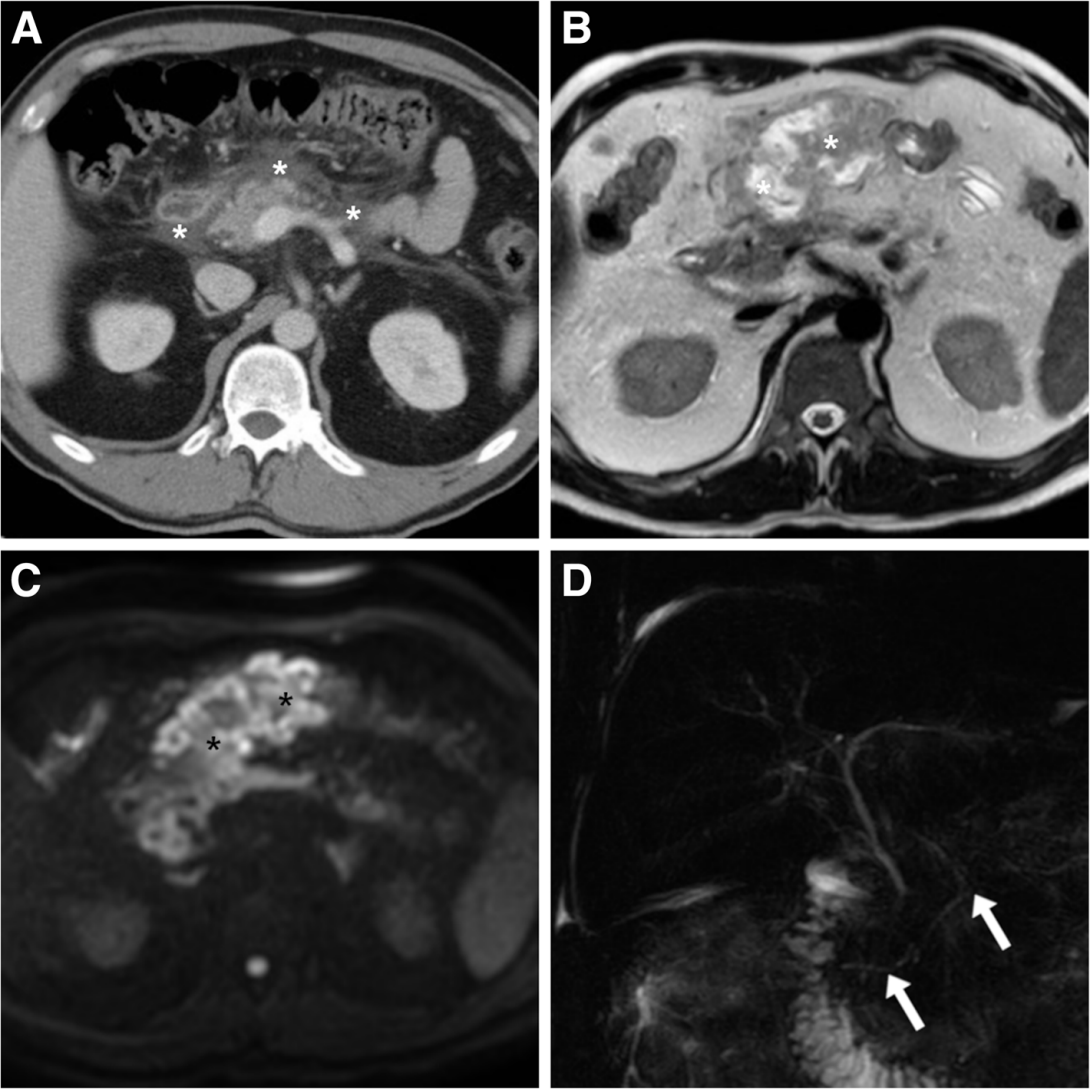
In a 44-year-old male with clinical and laboratory features consistent with mild AP, early contrast-enhanced MDCT (**a**) showed preserved pancreatic enhancement and mild peripancreatic oedema and fluid (*). A week later, MRI including T2-weighted (**b**) and diffusion-weighted (DW, **c**) showed development of a vast heterogeneous region of peripancreatic fat necrosis (*). Additionally, MRCP (**d**) showed pancreas divisum (MPD indicated by arrows draining into the minor papilla) as the underlying cause of AP. Afterwards, the patient had MPD stenting (not shown) through the Santorini duct to prevent recurrence of AP

Aiming to provide a consistent terminology, in 2012, the updated version of the Atlanta classification differentiated between interstitial oedematous pancreatitis (IEP) and necrotising AP (N-AP), the latter characterised by the development of necrosis in either the pancreas or peripancreatic tissue. At contrast-enhanced MDCT or MRI, parenchymal necrosis is heralded by “patchy” (Fig. 1a) or ample regions (Fig. 2a, b) of diminished or absent enhancement, quantified as < 30%, 30–50% and over 50% in the classical Balthazar CT severity index (CTSI) and its modified version which also considers extrapancreatic complications. Since the loose pancreatic capsule does not impede the spread of inflammatory changes, necrosis of peripancreatic fatty tissue (Fig. 3) is even more common [5–7].

In 30 to 50% of patients with AP, fluid collections develop secondary to either fluid leakage or liquefaction of tissue necrosis. As a result, respectively after IEP and N-AP, acute peripancreatic fluid collections and post-necrotic collections develop in or near the pancreas and frequently extend to the lesser sac, mesentery and anterior pararenal space. APFC contain sterile fluid with corresponding CT hypoattenuation (below 30 Hounsfield units) and homogeneous fluid-like MRI signal intensity. Both entities may either resolve within weeks or persist. Four weeks after the initial bout of AP, “mature” collections which have become organised within a radiologically perceptible wall are termed pseudocysts (after IEP) and walled-off pancreatic necrosis (WOPN) after N-AP, respectively. Since most APFC tend to resolve spontaneously, the majority of persistent pancreatic and/or peripancreatic collections are WOPN that characteristically show heterogeneous, non-enhancing content reflecting the presence of debris (Fig. 1). Owing to its superior soft-tissue contrast, MRI allows confident differentiation of pseudocysts from WOPN by visualising the internal heterogeneity with T2-hypointense necrotic debris (Fig. 1d) [5, 6].

**Fig. 4:**
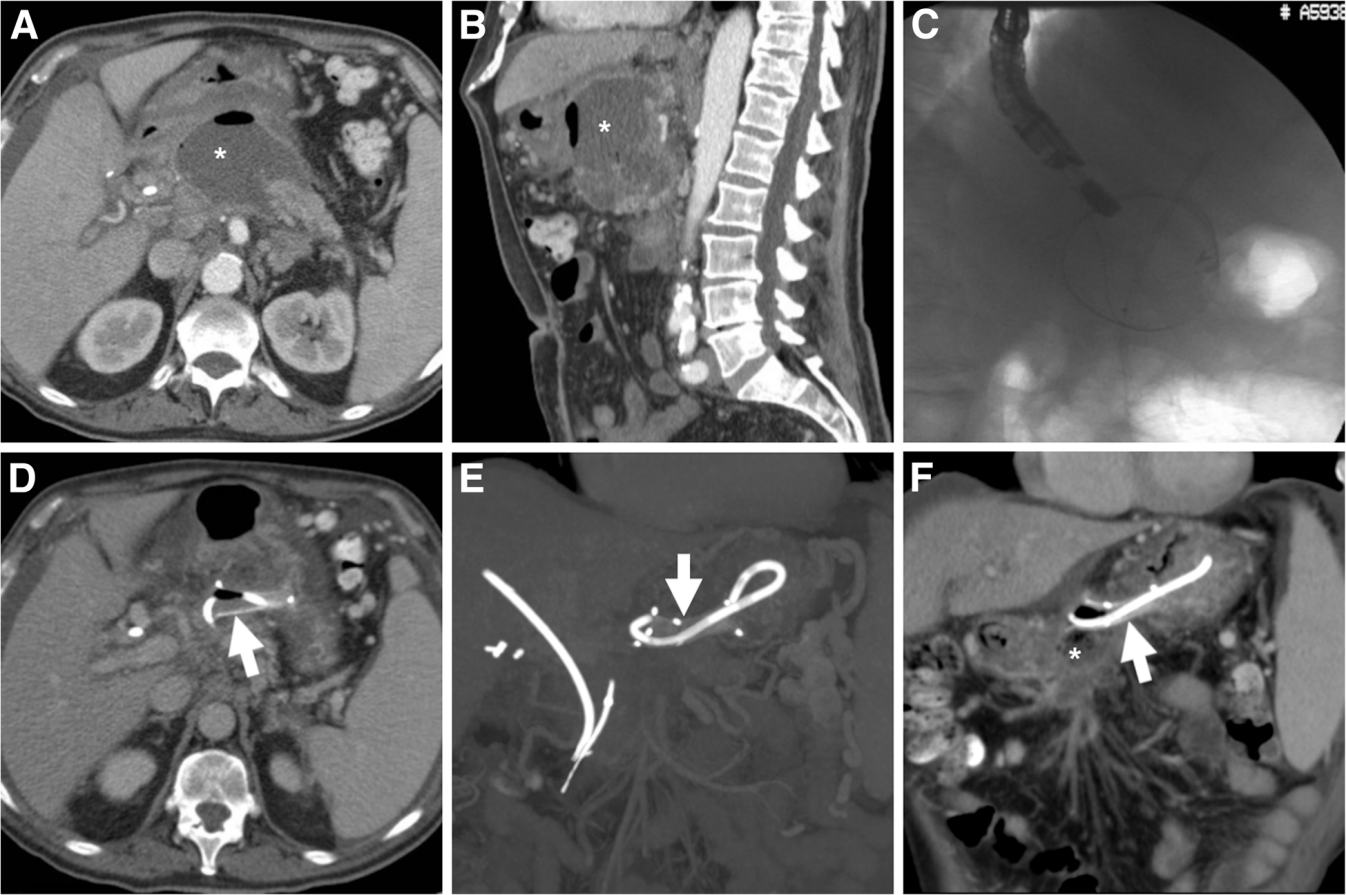
In a 67-year-old male with history of AP, following ineffective trans-duodenal drainage and stent loss, MDCT (**a**, **b**) showed reappearance of a fluid-containing collection (*) in the lesser sac with some nondependent air, suspicious for superinfected WOPN. Note biliary stent in place. Transferred to our hospital, endoscopic retreatment included cystogastrostomy and necrosectomy (**c**) with positioning of a plastic pigtail through the metal stent (thick arrows) in follow-up MDCT (**d**, **e**), a MPD stent (in maximum-intensity projection MIP E) and achieved rapid decrease of the collection. Ultimately, the metal stent was lost and the distal tip of the pigtail fell outside the incompletely collapsed collection (* in **f**)

Outside the setting of AP, analogous collections may develop after surgery or traumatic injury to the pancreas. Any collection may become infected, although this most commonly occurs in WOPN. The worrisome superinfection is heralded by consistent clinical and laboratory features, by increasing thickness and enhancement of its walls, and by development of internal gas without previous procedures (Fig. 4) [5, 6].

Additionally, MRI including diffusion-weighted imaging (DWI) improves the diagnostic accuracy to detect infection over CT with easy recognition of bright signal particularly at the periphery. According to Borens et al., abscesses show significantly lower apparent diffusion coefficient (ADC) measurements compared to non-infected collections, resulting in 67% sensitivity and 96% specificity using ADC cut-off values of 1012–1090 × 10–3 mm^2^/s [8, 9].

Finally, in the setting of AP, the routine acquisition of MRCP sequences (Fig. 1f) provides evaluation not only of causative gallstone disease but also of the pancreatic ductal system, specifically to look for congenital variants which may underlie AP such as pancreas divisum (Fig. 3) and for discontinuities of the MPD which correspond to a diagnosis of disconnected pancreatic duct syndrome (DPDS). Increasingly recognised after N-AP or pancreatic trauma, the latter is defined by MPD rupture with lost continuity between viable pancreas and the gastrointestinal tract, thus leading to leakage of pancreatic secretions, amylase-rich peritoneal and pleural fluid and formation of internal and external pancreatic fistulas. Coexistent DPDS is reported in up to 16% of patients with WOPN, but is often recognised late (2 to 9 months) after the initial N-AP bout in patients with non-resolving collections despite ineffectual percutaneous, endoscopic or surgical procedures.

Traditionally, the hallmark of DPDS was demonstration of MPD discontinuity at the neck (58% of cases), body-tail (23%) or mid-body (19%) during endoscopic retrograde cholangiopancreatography (ERCP). Extravasation of contrast medium injected during ERCP at the site of ductal disruption was reported in up to half of patients. Nowadays, DPDS (Fig. 2) should be diagnosed at cross-sectional imaging, based on the combination of (a) post-necrotic collection of variable size along the expected course of the MPD; (b) the presence of viable, enhanced pancreatic parenchyma distally to the collection; and (c) discontinuity of MPD on MRCP images. The key MRCP pitfalls are incomplete ductal disruption and false positives from superimposition or extrinsic compression by a collection. Unfortunately, despite most patients had several CT studies, DPDS is often missed at primary CT interpretation, thus contributing to the diagnostic delay [10–12].

Additionally, in the past decade, some centres investigated the use of secretin-enhanced MRCP in late stages of AP and described its value in improving delineation of MPD anatomy and disruptions. Additionally, this technique provides functional information regarding pancreatic fistulas, by showing ductal leakage and fluid accumulation around the pancreas or within collections, and may be reserved for complex or equivocal situations [10, 13].

#### Complications of chronic pancreatitis

**Fig. 5:**
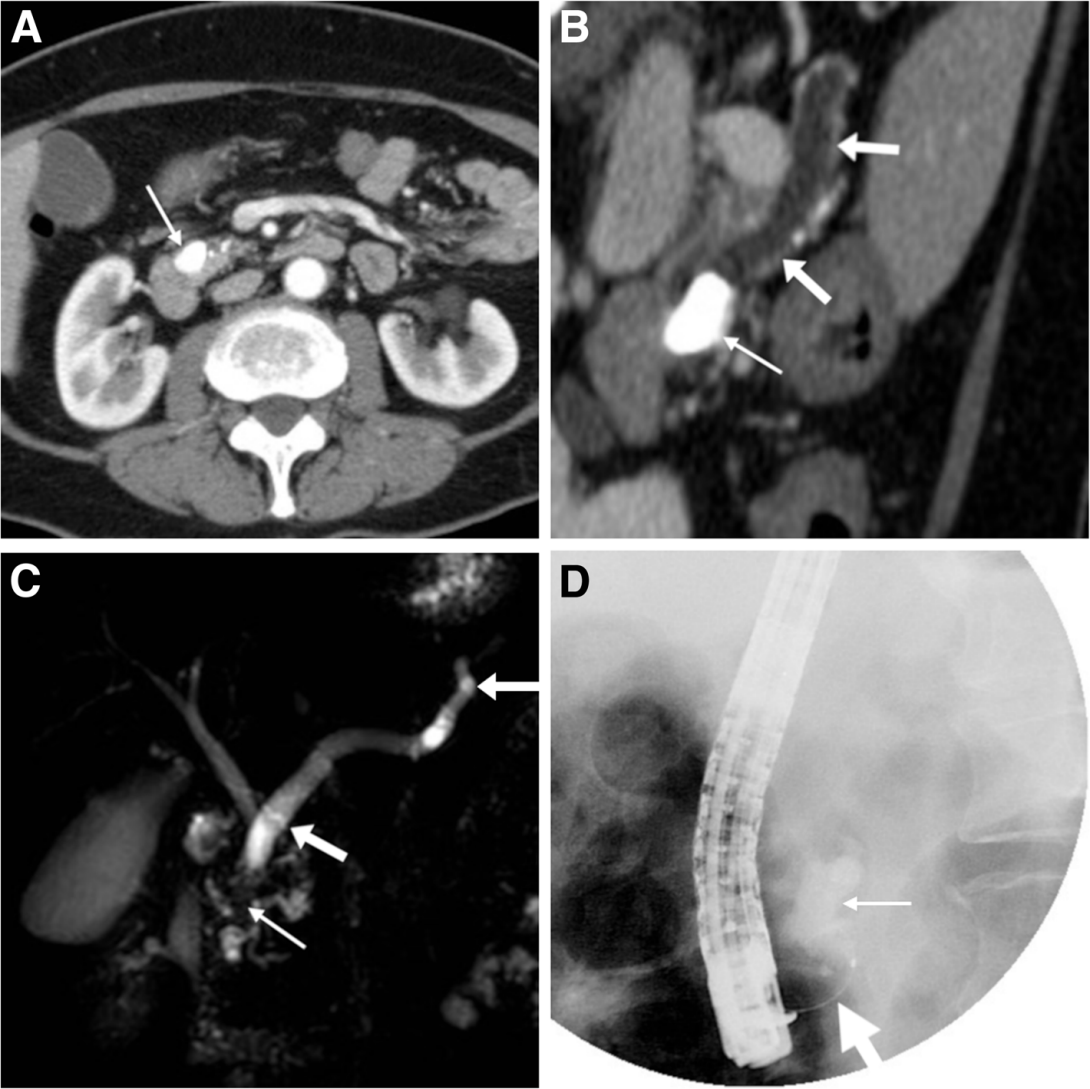
In a 63-year-old female with idiopathic chronic pancreatitis (CP) and parenchymal thinning long neck, body and tail of the pancreas, contrast-enhanced MDCT (axial image **a**, oblique reformation along MPD in **b**) showed diffusely dilated (maximum caliber 9 mm) MPD (arrows) upstream to a distal, densely calcified stone (thin arrows) measuring approximately 1.5 cm. MRCP (**c**) confirmed large intraductal stone as signal void (thin arrow) at distal MPD causing upstream obstruction. Endoscopic fragmentation (**d**) was unsuccessful due to impeded passage of guidewire, so the patient underwent extracorporeal shock wave lithotripsy (adapted from Open Access ref. no. [28])

**Fig. 6:**
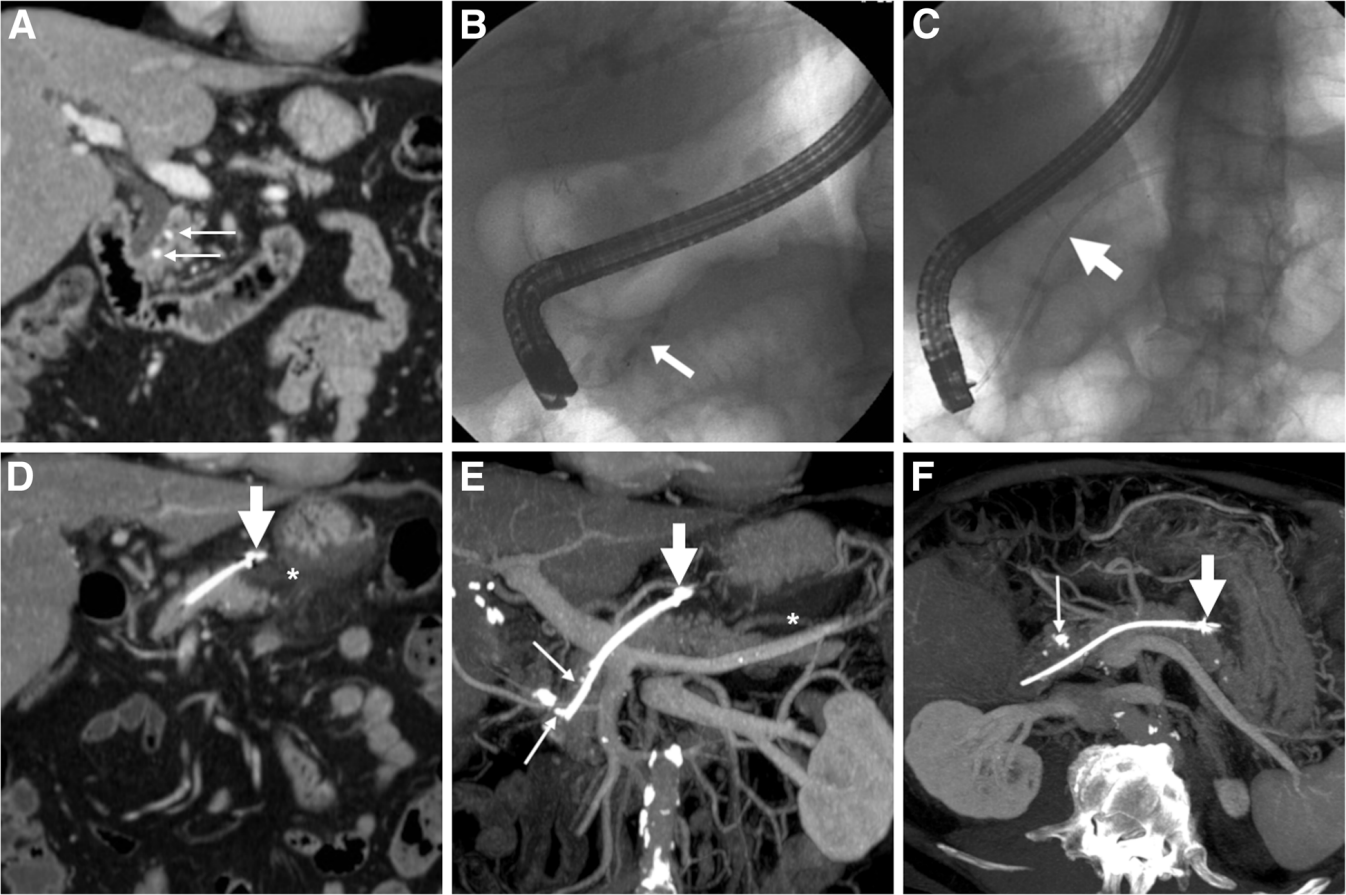
In a 68-year-old male with CP, oblique-coronal contrast-enhanced MDCT image (**a**) showed two tiny calcifications (thin arrows) at the cephalic tract of the MPD, a finding confirmed during endoscopic retrograde cholangiopancreatography (ERCP, **b**—MPD indicated by arrow) and treated by positioning of a long pancreatic stent (thick arrow in **c**). Repeated MDCT (**d**–**f**) showed the distal end of the MPD stent (thick arrows) to be located outside the pancreatic gland. Note that excellent depiction of stent (thick arrows) and burden of calcifications (thin arrows) by MIP reconstructions (**e**, **f**). Despite clinical improvement, the stent had to be removed

In patients with chronic pancreatitis (CP), MRCP is now established as the preferred technique for non-invasive visualisation of ductal changes such as dilatation, irregularities and strictures, as it (unlike ERCP) visualises the ductal system upstream to an obstruction. At MRCP, intraductal stones may be diagnosed as filling defects surrounded by static fluid within dilated ducts (Fig. 5). MDCT has a complementary role, since it better depicts calcifications that are often barely or not visible at MRI. Classically associated with full-blown CP, calcifications vary in size from punctate to centimetric and progressively accumulate over the course of the disease. The pancreatic burden of calcifications may be depicted by reconstructing maximum-intensity projection (MIP) images (Fig. 6e, f). Additionally, oblique-coronal and curved planar reformations along the MPD course help to discriminate parenchymal calcifications from intraductal stones (Figs. 5 and 6), the latter involved in the pathogenesis of the intraductal hypertension that ultimately leads to progressive tissue ischaemia, periductal fibrosis and parenchymal atrophy [14].

### Endoscopic cystostomy

#### Indications, technique and results

In the past, symptomatic pseudocysts and WOPN were managed by surgical cystogastrostomy, cystoduodenostomy or cystojejunostomy plus necrosectomy as required. However, open surgery is burdened by considerable morbidity, prolonged hospitalisation and non-negligible mortality; thus, surgeons progressively attempted the laparoscopic approach. The last decade witnessed a paradigm shift in the management of post-AP collections, with minimally invasive endoscopic cystostomy (ECS) and necrosectomy becoming the preferred treatment. Traditionally, indications for operative treatment included persistent collections measuring at least 6 cm in size and causing pain, discomfort, jaundice or gastric outlet obstruction. Nowadays, management is increasingly proactive due to awareness of risk of possible further complications such as superinfection, haemorrhage and rupture. According to the latest European Society of Gastrointestinal Endoscopy (ESGE) guidelines, the key indication for invasive intervention is suspected or proved infected necrosis. The choice between endoscopic and percutaneous drainage should rely on the WOPN location and local expertise. Selected cases of large retroperitoneal abscess collections may still require CT-guided drainage via posterior approach. Other indications for intervention include (a) progressively enlarging collections; (b) symptomatic WOPN, such as those causing gastric outlet obstruction or obstructive jaundice; and (c) clinical deterioration or failure to improve on conservative (medical) management [2, 15–18].

**Fig. 7:**
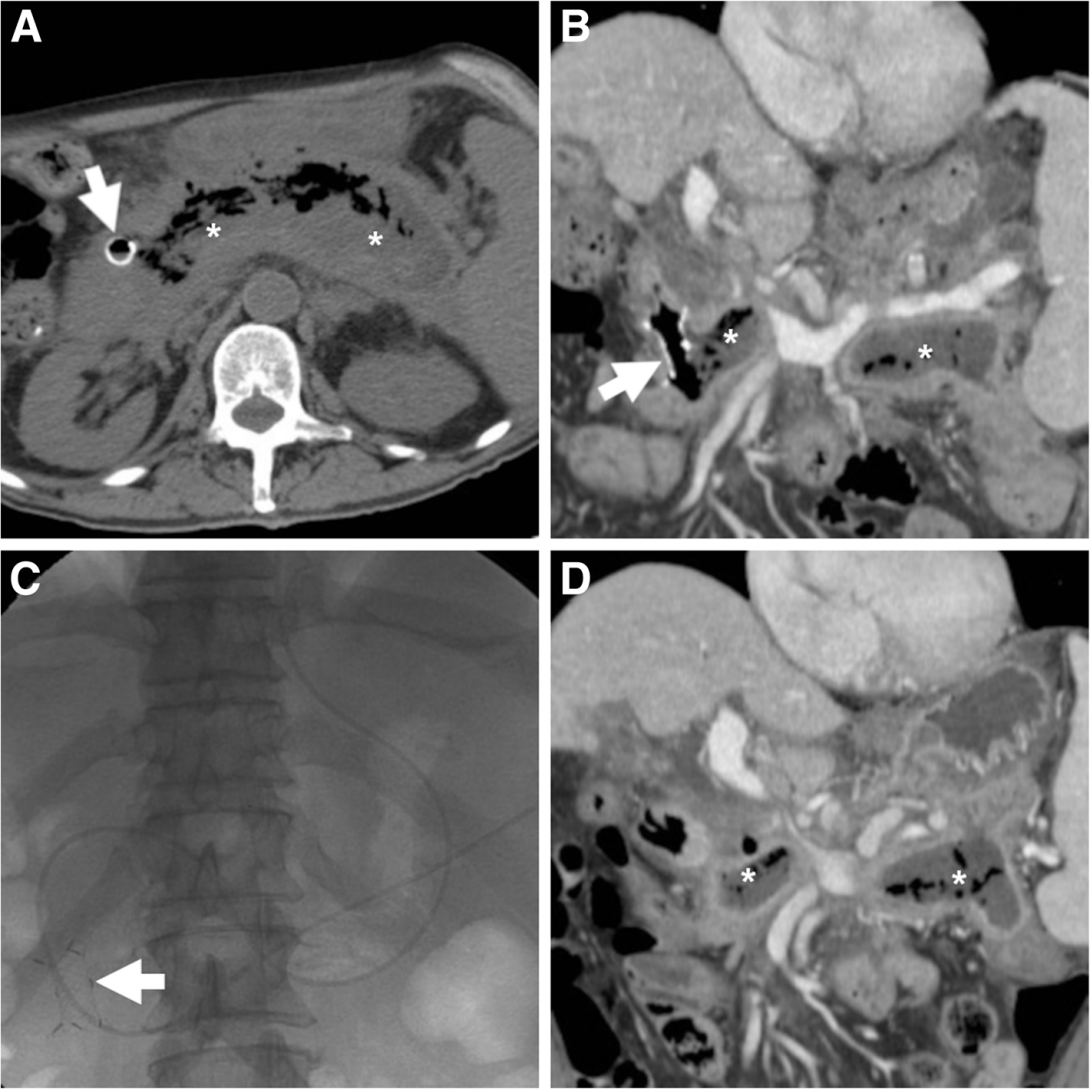
Post-procedural unenhanced (**a**) and post-contrast (**b**) MDCT in an elderly 81-year-old male following trans-duodenal endoscopic cystostomy (ECS) showed communication between duodenum and WOPN (*) via a metal stent (thick arrows), and development of air within the treated collection. Necrosectomy was then performed via nasobiliary drain through the stent (thick arrow in **c**). Follow-up MDCT (**d**) showed loss of the stent and partial decrease of the WOPN (*)

**Fig. 8:**
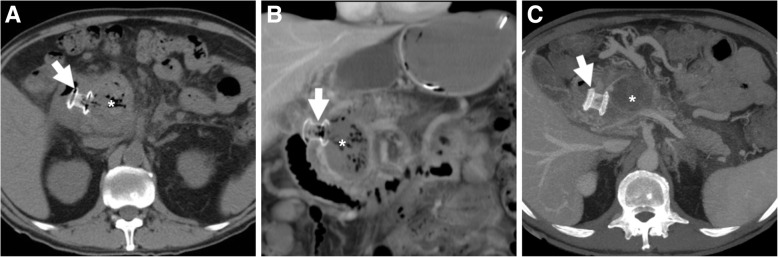
Unenhanced (**a**) and post-contrast (**b**, **c**) MDCT images in a 78-year-old male showed placement of a saddle-shaped lumen-apposing metal stent (thick arrows) at the site of ECS at proximal duodenum, best visualised using MIP reconstruction (**c**)

Preferably performed under EUS guidance to avoid varices along the intended track, ECS involves endoscopic puncture of the collection, creation of a fistulous tract with either duodenum (Figs. 7 and 8) or stomach (Figs. 2 and 4) using a guidewire, balloon dilatation and deployment of stents to facilitate drainage of the content into the gastrointestinal lumen. Both plastic (pigtail) and metal stents are considered effective and safe, but the latter may offer and advantage in WOPN and infected pseudocysts. The novel fully covered lumen-apposing metal stents (Fig. 8) are specifically saddle-shaped designed with bilateral flanges, to provide lumen-to-lumen anchoring, improve drainage and decrease the risk of migration and leakage. After accessing the WOPN using a gastroscope, debridement of the necrotic material (necrosectomy, Figs. 2, 4 and 7) is performed using biopsy forceps and snares. At our hospital, endoscopists most usually place a metal stent to keep the ECS patent, then perform trans-stent necrosectomy and finally place one or two pigtails through the metal stent (Fig. 2d). Furthermore, during the ECS procedure, visualisation of the MPD by ERCP is generally warranted, to avoid missing the diagnosis of coexistent DPDS.

EUS-guided ECS achieves technical and clinical success in 97% and 91% of cases, respectively, has lower morbidity and allows faster recovery compared to surgery, which is currently reserved for those patients with unfavourable anatomy [2, 16–19].

#### Cross-sectional imaging after endoscopic cystostomy

Post-ECS imaging, most usually with MDCT, is usually necessary to assess position of devices, efficacy of treatment and possible development of complications. In our experience, performing MIP reconstructions is useful for depicting shape, position and features of ECS stents (Figs.4 and 8), which should have the proximal tips in the stomach and the distal tips in the collection being treated. After ECS, introduction of air within the treated collection is an expected finding and should not be reported as worrisome for superinfection [3].

**Fig. 9:**
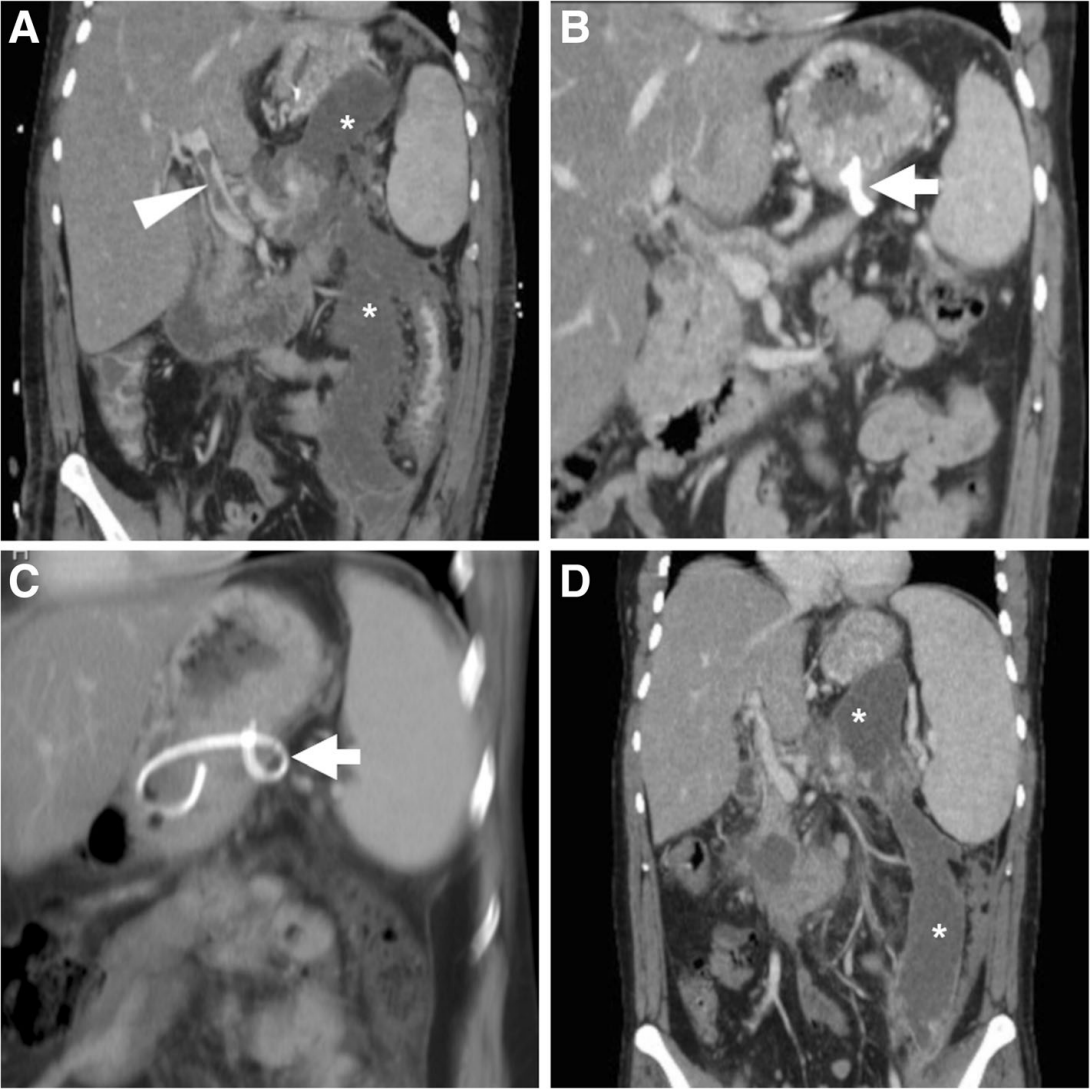
In a 57-year-old male with severe AP, initial MDCT (**a**) showed thrombosis of the portal vein (arrowhead) and extensive, confluent acute post-necrotic collections (*). Weeks later, repeated MDCT (**b**, **c**) showed complete regression of collections following ECS performed using only a pigtail stent (thick arrows). However, after stent loss WOPN (* in **d**) ultimately recurred

The expected, favourable outcome is progressive decrease in size of the pseudocyst or WOPN (Figs. 4, 8 and 9). After successful ECS, migration of the stent into the stomach and transit to the bowel may ultimately occur (Fig. 7). Sometimes, internal drainage stops when the distal end of the stent(s) fall outside the progressively collapsing collection (Fig. 4f), a finding which may require repositioning. Conversely, in exceptional cases, the entire stent may be internalised within the collection [2, 3, 18].

Failure of ECS is heralded at MDCT by insufficient decrease of the treated collection (Fig. 7) or secondary re-accumulation of fluid after initial drainage (Fig. 9d): WOPN recurrence from stent dislodgement or occlusion occurs in 5.7–17.7% of patients and may require retreatment.

Post-ECS complications may develop after a mean 15% (range 0–50%) of procedures and mostly include bleeding from injured pancreatic vessels, superinfection and stent migration. Risk factors for complications include too early ECS after initial AP, and treatment of WOPN or abscesses (risk 40%) compared to pseudocysts (9.2%) [2, 16–19]. Similarly to the conventional ERCP setting, complications are diagnosed on the basis of a combination of intraprocedural events, clinical symptoms (sudden or worsening abdominal pain, fever), hemodynamic impairment, elevated leukocyte count or acute phase reactants developing hours or days after ECS. Whereas gas results from the iatrogenic cysto-gastric communication, infection is generally suggested by increasing thickness and enhancement of the enhancing wall. Haemorrhage is heralded by hyperattenuating fresh blood in the duodenal lumen and warrants an additional arterial-phase CT acquisition to detect possible active contrast extravasation indicating ongoing bleeding [3, 20].

### Endoscopic interventions on the main pancreatic duct

#### Indications, technique and results

Performed during either conventional transpapillary ERCP or using EUS guidance, long-term MPD stenting is indicated to manage DPDS, benign (such as those from CP), postsurgical and selected malignant strictures [1, 15, 21]. Furthermore, in high-risk patients prophylactic MPD stenting is also indicated to prevent hyperamylasaemia and iatrogenic AP after ERCP [22].

Recognising and treating DPDS is paramount since it is associated with increased AP severity, recurrence risk and long-term complications, frequent failure of conservative management and low rates of resolution after drainage compared to post-AP peripancreatic fluid collections. In the past, surgery, by either redirecting secretions into the digestive tract (such as via pancreatic anastomosis to a Roux-en-Y jejunal limb) or resecting the viable disconnected pancreatic segment (most often with a distal pancreatectomy), was burdened with high morbidity and mortality. Nowadays, endoscopic stenting of the MPD (Fig. 2) is increasingly recognised as the best option (successful in 75% of patients) to manage DPDS, coupled with EUS-guided transgastric ECS of associated pseudocysts or WOPN [23, 24].

In the setting of CP, MPD stenting (Fig. 6) of dominant strictures is beneficial to decrease the intraductal hypertension which is involved in the pathogenesis of CP-related pain. Before stenting, pancreatoscopy-guided fragmentation and removal of intraductal stones may be performed. Small calculi (< 5 mm in size) may be extracted using baskets or trawls through endoscopic sphincterotomy. Prior to endoscopic retrieval, larger or impacted stones should be fragmented using extracorporeal shock wave lithotripsy or, more recently, endoscopic electrohydraulic lithotripsy (Fig. 5). Extracorporeal shock wave lithotripsy remains the first-line therapy for large obstructive MPD stones. Variable (in the range 50–80%) clinical success rates have been reported in endoscopic management of CP [1, 21, 25].

After MPD cannulation, stents are inserted using a guidewire. Diameter and length of stents should be chosen according to ductal configuration, site and features of the stricture or discontinuity: smaller ones (5 to 7 French) are used in the absence of duct dilatation, larger (10 French) stents are required when upstream dilatation is present. Plastic pancreatic stents made of polyethylene have side holes to allow drainage of pancreatic juice from lateral branches, and remain the mainstay measure for managing benign strictures. However, the advent of fully covered metal stents increasingly allows an effective treatment with less need for stent replacement [1, 21].

#### Cross-sectional imaging after pancreatic ductal stenting

**Fig. 10:**
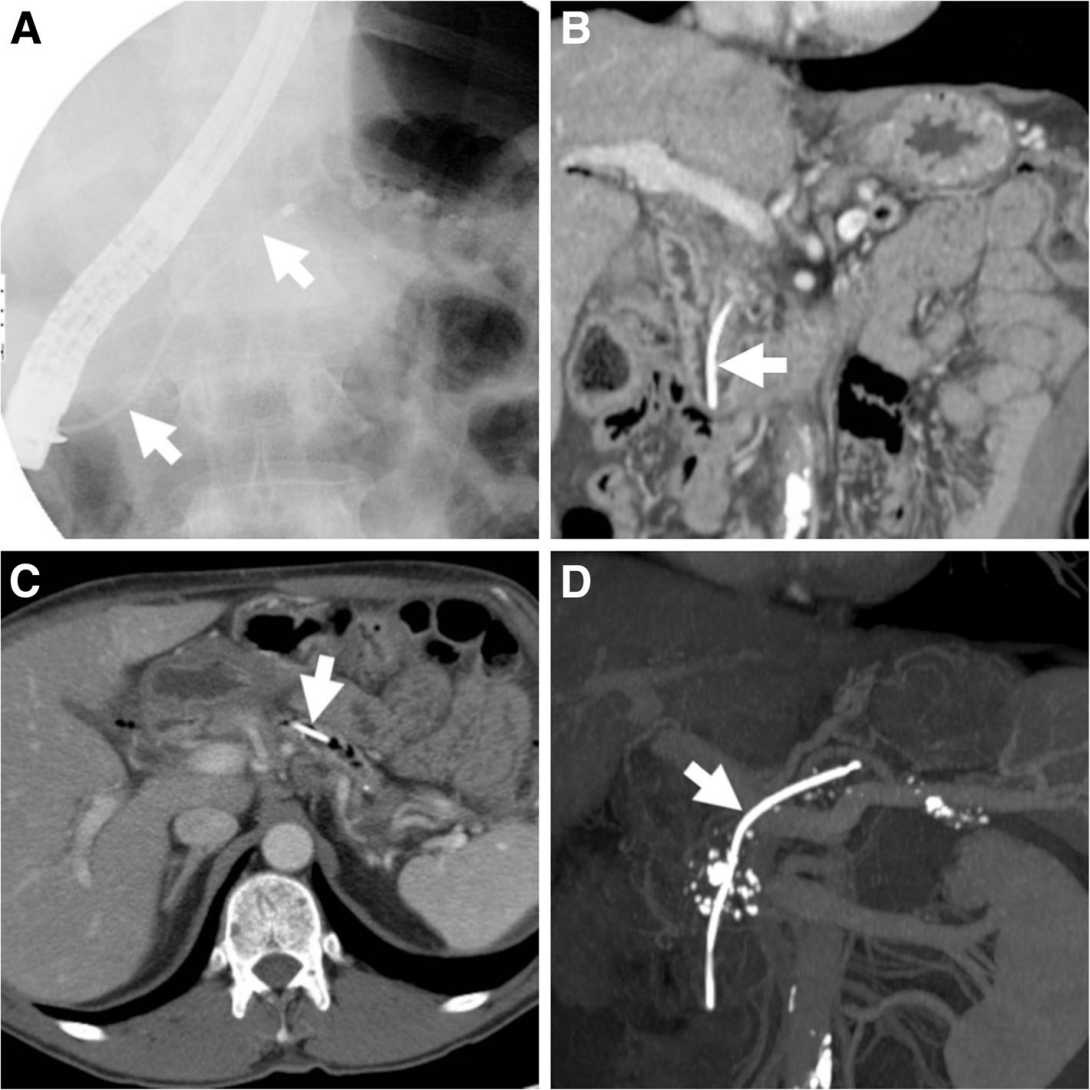
In a 59-year-old male with CP, a long stent (thick arrow in **a**) was placed to relieve hypertension of the MPD. Post-procedural MDCT (**b**, **c**) showed correctly placed stent (thick arrows) from near the Vaterian ampulla through the pancreatic midbody, without signs of complications. MIP reconstruction (**d**) confirmed stent integrity (thick arrow) and visualised entity and distribution of parenchymal pancreatic calcifications

Following MPD stenting, MDCT is often requested to assess the postoperative status and rule out possible complications. Using appropriate multiplanar image review, MDCT (Figs. 6 and 10) effectively depicts the stent’s position and course, including the pancreatic burden of calcifications on MIP reconstructions. Despite EUS guidance and increasing experience, advanced endoscopic interventions on the pancreatic ductal system remain technically challenging procedures with a non-negligible morbidity. Complications occur in roughly 20% of patients (up to 55% when stent migration is included), without significant differences between traditional ERCP and EUS-guided technique. Stent migration is by far the commonest adverse event with both plastic and metal stents, unrelated to patient factors, aetiology, location and stent features. Stent malpositioning (Fig. 6) and fracture are relatively less common. In descending order of frequency, other complications include abdominal pain, AP, haemorrhage, abscess formation and perforation. Whereas some retroperitoneal air may be observed in early MDCT studies following operative ERCP, the latter is suggested by extensive emphysema dissecting through the retroperitoneum, appearance of pneumoperitoneum or new periduodenal fluid collection [1, 20, 21, 26].

## Conclusion

Novel interventional techniques have put endoscopy at the forefront of management of complications of acute and chronic pancreatitis. Aiming to provide radiologists with an adequate familiarity with these procedures, this pictorial essay reviewed the related pre- and post-procedural cross-sectional imaging features. As recognised by the ESGE guidelines, the use of MRI is particularly helpful in patients in whom invasive intervention for post-AP collections, possible infection and/or DPDS is being considered [15].
